# A New Frontier in Exciton Transport: Transient Delocalization

**DOI:** 10.1021/acs.jpclett.2c01133

**Published:** 2022-07-20

**Authors:** Alexander J. Sneyd, David Beljonne, Akshay Rao

**Affiliations:** † Department of Physics, Cavendish Laboratory, University of Cambridge, Cambridge CB3 0HE, United Kingdom; ‡ Laboratory for Chemistry of Novel Materials, University of Mons, Mons 7000, Belgium

## Abstract

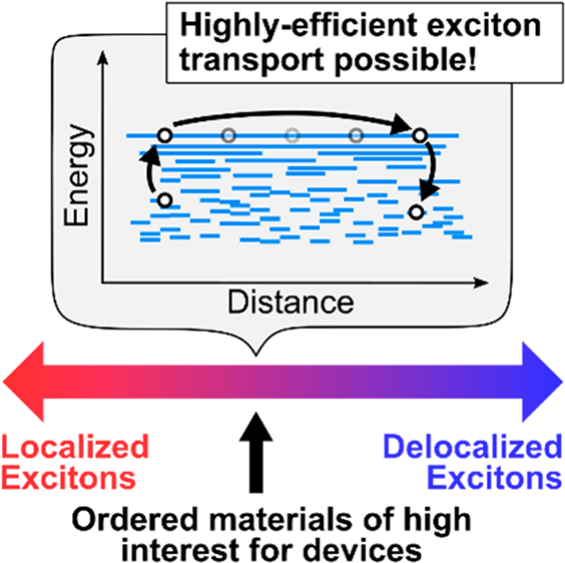

Efficient exciton transport is crucial to the application of organic semiconductors (OSCs) in light-harvesting devices. While the physics of exciton transport in highly disordered media is well-explored, the description of transport in structurally and energetically ordered OSCs is less established, despite such materials being favorable for devices. In this Perspective we describe and highlight recent research pointing toward a highly efficient exciton transport mechanism which occurs in ordered OSCs, transient delocalization. Here, exciton–phonon couplings play a critical role in allowing localized exciton states to temporarily access higher-energy delocalized states whereupon they move large distances. The mechanism shows great promise for facilitating long-range exciton transport and may allow for improved device efficiencies and new device architectures. However, many fundamental questions on transient delocalization remain to be answered. These questions and suggested next steps are summarized.

The transport of energy via excitons is an essential process underlying the operation of light-harvesting devices based on organic semiconductors (OSCs), such as organic photovoltaics, photodetectors, and photocatalytic systems. This is because it allows excitons photogenerated in the bulk of an OSC to reach charge-generating heterojunctions. Ideally, OSCs would be able to transport excitons over distances comparable to the material thicknesses that allow for the full absorption of light (>200 nm). However, the majority of OSC materials developed to date exhibit short-range and slow transport, particularly in the case of device-relevant materials, and exciton diffusion lengths (*L*_D_) are generally limited to <10 nm with associated diffusion constants (*D*) on the order of 10^–3^–10^–4^ cm^2^/s.^1−3^ Such performance places stringent constraints on the design of organic devices; for instance, it necessitates the use of nanoscale bulk heterojunctions where donor and acceptor OSCs are tightly intermixed together. This architecture means that photogenerated excitons need only travel short distances to reach the heterojunction to be split into free charges, but it also compromises other aspects of the device, such as charge extraction, stability, and reproducibility.^4,5^ Hence, the ability to engineer long-range transport phenomena in device-relevant OSCs has remained an outstanding challenge for several decades, which if achieved could allow for higher device power conversion efficiencies and new design architectures.

Central to the goal of engineering long-range transport is developing a better understanding of the fundamental physics of exciton transport itself, as this will allow for effective design rules. Our current understanding of exciton transport is heavily dominated by the idea that excitons in OSCs are localized and hop incoherently from site to site via Förster resonance energy transfer (FRET).^1−3^ However, for more ordered OSCs, excitons can be delocalized over several individual chromophores/molecules, and the FRET model is no longer an accurate description of the underlying physics.^6−11^ In these cases, contributions from the delocalized nature of the excitons are expected,^6−8^ but the exact details of exciton transport have not been fully established. Herein, we highlight recent findings which elucidate a general transport mechanism in the intermediate regime where OSCs are ordered and excitons can be partially delocalized. Not only does this new mechanism—transient delocalization—force us to revisit our basic picture of exciton dynamics in OSCs, but it is also shown to be highly effective for transport, and further improvement and optimization could be transformative for devices based on OSCs.

*A Tale of Two Extremes: FRET and Coherent Transport*. Part of the reason FRET has dominated our understanding of exciton transport is because it does indeed provide an accurate description of transport when excitons are localized.^1,12^ Most of the OSCs used in devices over the last several decades, such as spin-coated polymeric thin films, support only highly localized excitons; that is, the excitons reside on a single chromophore. This localization is a consequence of the high static and/or energetic disorder pervasive in OSC films made from common preparation processes such a spin-coating or thermal evaporation. The localization is furthermore exacerbated by several factors which are inherent to most OSCs, such as high dynamic disorder due to strong exciton–phonon couplings and the associated high reorganization energies (the energy barrier for exciton transfer) together with weak electronic couplings between chromophores. These factors work together to destroy the long-range coherence of the exciton wave functions, preventing excitons from moving in a wavelike manner as they would do in a perfect crystal. Instead, the localized excitons “hop” incoherently between chromophores, with this hopping chiefly mediated by two fundamental interactions within the OSC: short-range exchange or superexchange (charge-transfer mediated) coupling, and long-range Coulombic (dipole–dipole) interactions. Note that the former interactions can be either through-space (e.g., between molecular building blocks) or through-bond (e.g., between chemically linked conjugated segments along a single polymer chain). In multichromophoric systems, exchange coupling drives Dexter energy transfer, which is the dominant mechanism for the transport of dark states (such as triplets) but is substantially slower than FRET for bright singlet excitons.^13^ This Perspective will not focus on Dexter transfer or on through-bond intrachain migration; however, the interested reader is directed to refs (14−16). The second interaction, the Coulombic dipole–dipole interactions between chromophores, leads to hopping that can be modeled by the FRET framework.^12^ Here, the rate of transfer, , from donor to acceptor is given by
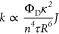
where  and  are the quantum yield and lifetime of the donor, respectively;  and  are the orientation and distance between the two chromophores, respectively;  is the refractive index; and  is the integral of the overlap between the donor’s emission and acceptor’s absorption.^5^ Unfortunately, organic materials typically exhibit large Stokes shifts with the emission substantially red-shifted from the absorption, and so  is low when the donor and acceptor are the same material. Furthermore, in the presence of energetic disorder, excitons will gradually tend to migrate to lower-energy donor sites, lowering  further.^17^ Other impeding factors include structural disorder (lowering κ) and the fact that short exciton lifetimes (τ) also reduce the available time with which the exciton can undergo FRET, balancing out any gains that might be had by increasing the rate of FRET (as *k* is inversely proportional to τ). The end result is that FRET-mediated exciton transport is relatively slow and short-range, typically with sub-10 nm exciton diffusion lengths.^1^

Given that highly localized excitons move via limited processes such as FRET (see Figure 1), we might then ask what happens when an exciton in an organic material becomes delocalized, perhaps through reducing dynamic disorder by lowering the temperature or through increasing electronic couplings throughout the material? In the most extreme case, as shown by Dubin et al.,^18^ excitons in a single isolated polydiacetylene chain at 10 K can be delocalized over tens of micrometers. To form this macroscopic coherent state from the excitons’ initial ∼1 μm starting excitation point, they move coherently. In other, less-idealized instances at room temperature, transport beyond the maximum rate allowed by FRET has been observed in highly ordered pristine nanostructures over distances of hundreds of nanometers.^6−8^ It has been speculated that coherence or coherent transport plays an important role as the states responsible for transport were shown to be delocalized—in one case over up to 10 molecules.^7^ Another interesting case is that of natural light-harvesting complexes (LHCs). Here, a large body of work has suggested that living organisms precisely arrange chromophores in ways that promote exciton delocalization and promote vibronic coherences that assist in energy transport.^19^

**Figure 1:**
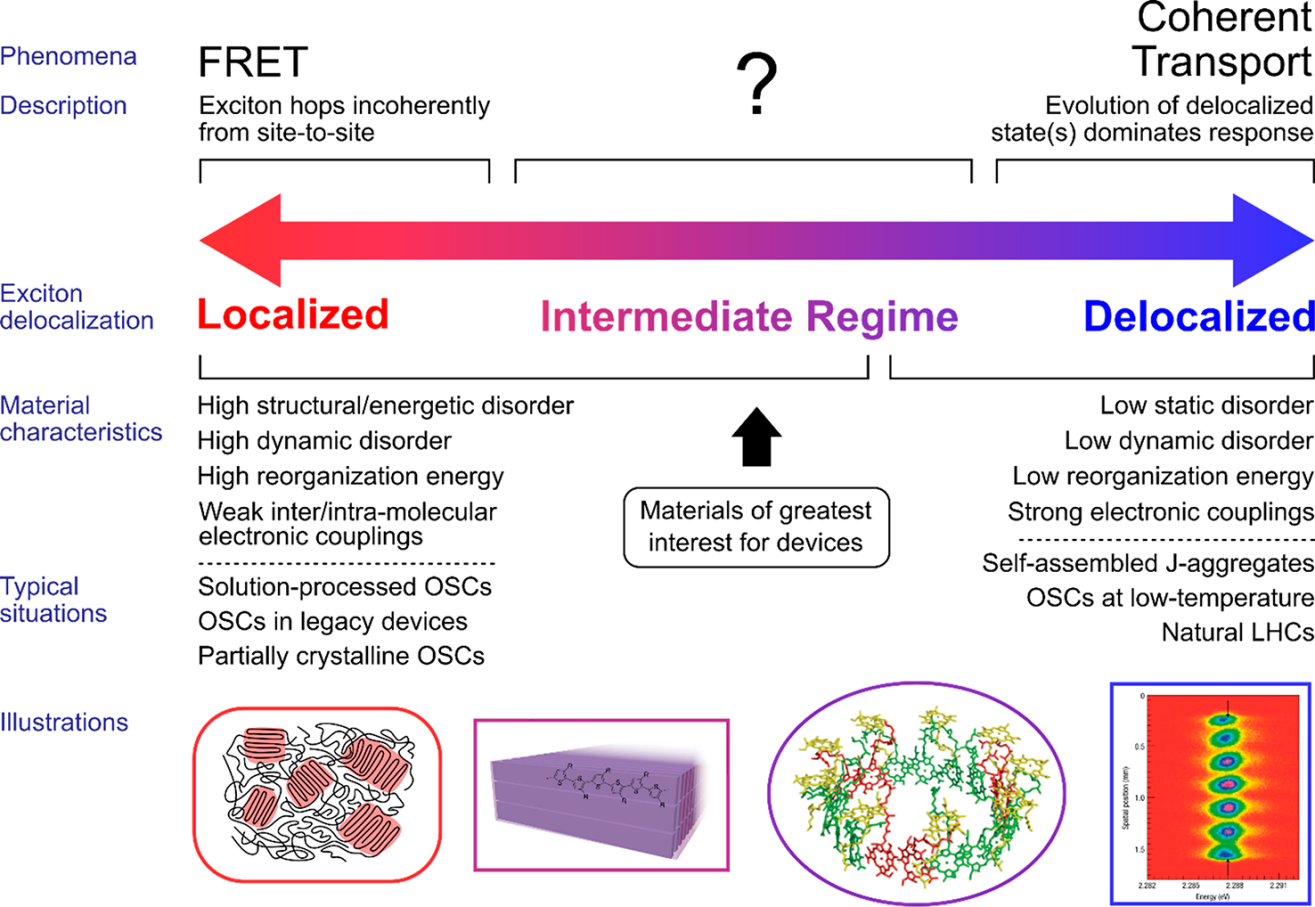
Schematic of knowledge gap on exciton transport in OSCs. On the left, when excitons are highly localized by a combination of high disorder and weak electronic couplings, transport primarily occurs via FRET. At the other extreme, on the right, where excitons are highly delocalized because couplings ≫ disorder, transport proceeds coherently. However, in the intermediate delocalization region, the way in which transport occurs largely remains a mystery. This region is also where many materials of high interest for devices are expected to fall as certain characteristics beneficial for devices, for example, high structural and energetic disorder, also promote delocalization. The bottom panel gives several illustrations of real-world examples of OSCs that exhibit excitons of varying levels of delocalization: (from left) a partially crystalline polymer film, aligned P3HT nanofibers, an LHC complex from Rps. Palustris, and the interfered emission from a cryogenically cooled polydiacetylene chain which shows macroscopic exciton coherence. Reproduced from ref (18). Copyright 2005 Nature Publishing Group.

The exact variables which control coherent transport are the subject of ongoing research; however, it is generally considered that coherent transport arises when the coupling between chromophores is comparable to or exceeds the energetic disorder.^9^ This presents a challenge as most OSCs, particularly those useful for device applications, typically do not exhibit large electronic couplings, and they also typically exhibit substantial static energetic disorder. Another important aspect to note is that coherent transport is a short-lived phenomenon at room temperature because of scattering off phonons. For example, rapid exciton transport (∼10 cm^2^/s) was observed in the first ∼500 fs in perylene-diimide thin films, before an order of magnitude slowdown in the observed transport in the next few picoseconds due to phonon interactions.^20^ This contrasts with incoherent processes such as FRET, which has the same transfer rate at all points of the exciton’s lifetime. Hence, while coherent transport is certainly an alluring prospect for devices, it has seen limited applicability because of the difficulty of engineering sufficient couplings and because phonons will inevitably introduce scattering at room temperature, preventing purely coherent motion over distances large enough to permit substantive device design changes (>200 nm).

*The Regime of Intermediate Delocalization?* Our current understanding is therefore that in the case of highly localized excitons, transport proceeds via FRET, a relatively slow process limited by factors like the large Stokes shift of OSCs. At the other extreme, delocalized states evolve coherently; however, engineering such phenomena is challenging in device-relevant materials, and it is only short-lived at room temperature. This raises a question of both fundamental and practical importance: how might exciton transport proceed in the intermediate regime where excitons are partially delocalized? Would such transport be efficient and useful for devices? As delocalization is a continuous (as opposed to a discrete) property, we might expect behavior combining elements of both FRET and purely coherent transport. Indeed, this appears to be the case for natural LHCs; for more information see the reviews in refs (19), (9), and (10). However, very little is known for the case of bulk OSCs, which differ from LHCs in several significant ways such as the types of molecules incorporated, the molecular packing, and the fact that bulk OSCs often consist of larger, compact, crystalline structures. This lack of knowledge is concerning because not only are devices predominantly built from bulk OSCs, but also the materials used in the most promising devices (which excel in terms of charge extraction, stability, and low open-circuit voltage) are often those that incorporate OSCs with high structural and energetic order—properties which should promote exciton delocalization.^21^

Given the importance of this area, we might ask what has impeded progress so far? The issue can be broken down into three key challenges. First, a great deal of synthetic control is needed to produce OSCs that have the requisite energetic and/or structural order, electronic couplings, or other characteristics needed to support transport phenomena beyond that of simple FRET. Second, the experimental observation of exciton transport itself is rather challenging, and most efforts have relied on indirect methods that exploit phenomena such as the exciton-annihilation-related shortening of exciton lifetimes or surface quenching as proxies for transport. Third, a theoretical understanding of exciton transport in these materials requires advanced methods that model the evolution of the exciton wave function quantum-mechanically and incorporate nonadiabatic behavior, i.e., the continual (and significant) exchange of energy between vibrational modes and excitons. Such models are complex and expensive as OSCs typically have large unit cells, large supercells need to be simulated, and many distinct phonons modes are present. However, for each of these challenges, recent advances provide great hope; supramolecular chemistry, particularly with the advent of living crystallization-driven self-assembly, now offers unparalleled opportunities to produce materials with very high energetic and structural order.^6,22−24^ The development of transient-absorption microscopy (TAM) now allows for the direct tracking of excited states with temporal and spatial-localization precisions of <10 fs and <10 nm, repectively.^25,26^ Finally, increasingly sophisticated (and scalable) methods have been developed, chiefly in the context of modeling charge transport in bulk OSCs.^27−30^ The methods have proved highly successful at reproducing the charge mobilities in a range of high-performing bulk OSCs,^28,31−33^ even at the intermediary crossover point between the classic charge transport mechanisms of hopping and band-like transport—a crossover not unlike the one already discussed here. Therefore, researchers are now well-positioned to tackle this pertinent problem of intermediary exciton transport phenomena.

Recent results have indeed shown great promise, hinting that OSCs that support semidelocalized excitons can also exhibit highly efficient transport. Examples of this include porphyrin nanotubes which supported partially delocalized excitons, with the delocalization extent ranging from about 1 to 10 molecules. TAM of the nanotubes revealed exciton diffusion coefficients of *D* = 3–6 cm^2^/s.^7^ Exciton-annihilation measurements on a similar system (cyanine dye aggregates) suggested a  as high as 50 cm^2^/s. Furthermore, oligomeric polyfluorene-based nanofibers, which support excitons localized on average on a single chromophore, exhibited a diffusion length of almost 300 nm with an associated  of 0.5 cm^2^/s.^6^ In each of these cases, FRET was unable to explain the observations as it predicted much lower diffusion rates. It is also important to note these experiments were conducted at room temperature, in contrast with the cryogenically cooled polydiacetylene system which exhibits delocalization over tens of micrometers.^18^ That exciton transport can still proceed so efficiently with only modest exciton delocalization is cause for excitement and provides a strong motivation for further investigation.

*The Transient Delocalization Mechanism*. This brings us to the most recent developments, where in 2021 we reported that highly ordered polythiophene-based nanofiber films (see Figure 2a), despite supporting excitons mostly localized on a single chain, exhibited highly efficient exciton transport, which we explained with a new *transient exciton delocalization* theory.^34^ In this study, TAM was used (see Figure 2b,c) to obtain a  of 1.1 cm^2^/s and an estimated  of 300 nm—values which are above the limit predicted by FRET and much higher than the typical values found for polythiophene films (10^–3^ cm^2^/s, and ∼10 nm).^35−37^ This performance was roughly on par with the other reports of efficient exciton transport.^6−8^ As an added benefit, the demonstration was now given for a compact OSC *film* with strong visible-light absorption. The behavior was rationalized by the exceptional long-range structural and energetic order of the nanofiber films, which was the result of the highly controlled epitaxial synthetic process used to create the nanofibers, living crystallization-driven self-assembly (CDSA). The transport in these systems was modeled using nonadiabatic molecular dynamics simulations, which used a mixed quantum–classical, crossing-corrected variant of the subspace surface-hopping algorithm, incorporating stochastic nonadiabatic transitions between different adiabatic potential energy surfaces. The methods implemented represent, to the best of our knowledge, the first attempt at utilizing state-of-the-art surface-hopping methods (typically used to quantitatively model charge transport) in the context of exciton transport. Analysis of the simulations revealed unique behavior, dubbed transient exciton delocalization, where excitons periodically exchange energy with vibrational modes to temporally access spatially extended states whereupon they move large distances.

**Figure 2:**
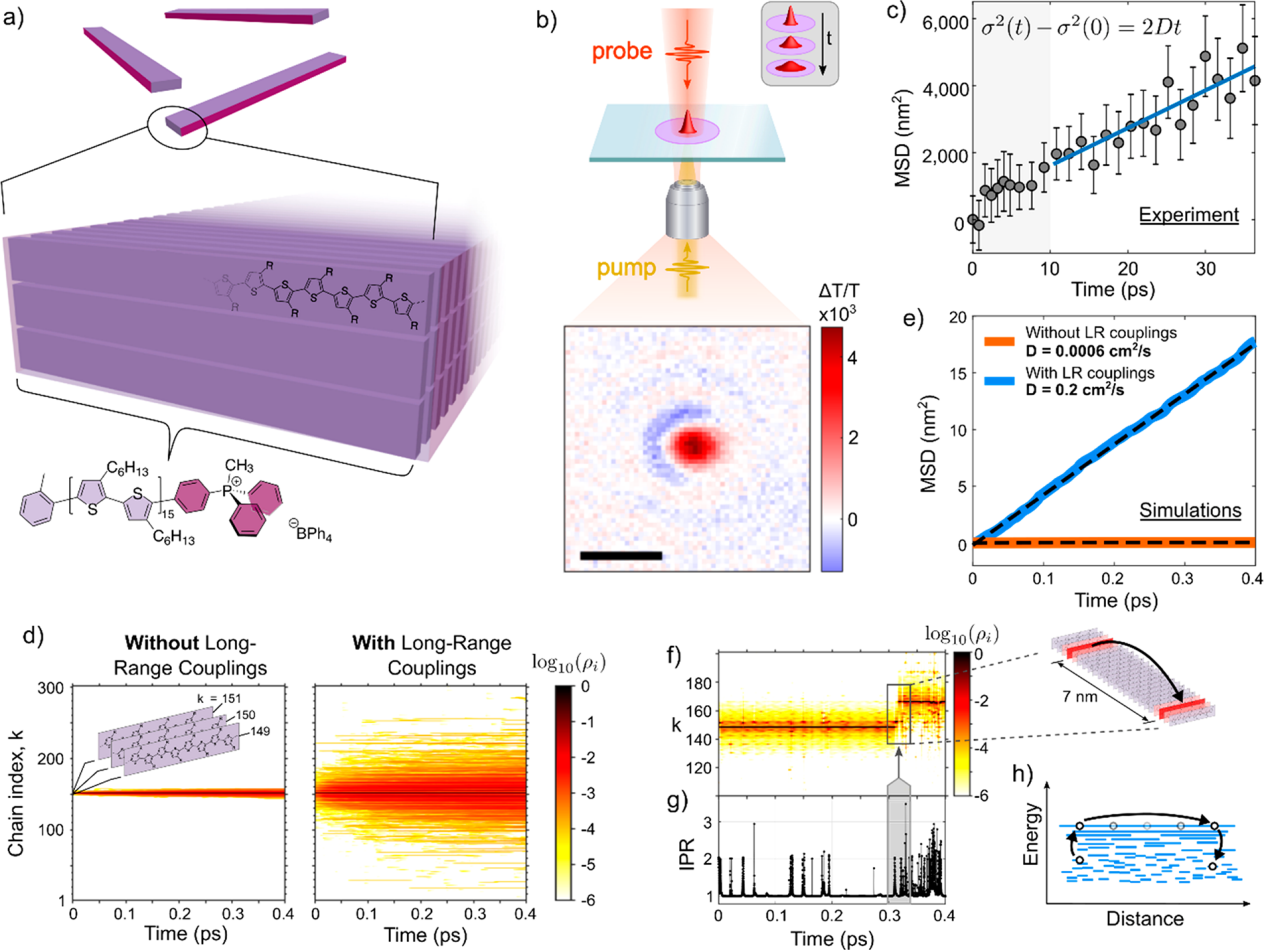
(a) System of study: P3HT-based nanofibers formed from living crystallization-driven self-assembly, where short unimer polymer strands are π-stacked together. (b) Schematic of transient-absorption microscopy experiment, with inset showing expansion of pump-generated exciton distribution over time. Scale bar is 1 μm. (c) Typical mean-square displacement (MSD) trace over time found by Gaussian fitting to distribution shown in panel b. Linear fit gives diffusion constant. (d) Total exciton density over time for 10 000 individual exciton trajectories, in two cases: one including only nearest neighbor couplings, and the other including all couplings. (e) Associated MSD found from panel d for each case, with linear fits. (f) Exciton density in representative exciton trajectory, along with (g) the associated inverse participation ratio (IPR) trace, which parametrizes the degree of delocalization. (h) Schematic of transient delocalization process, where excitons occasionally jump up to higher-lying delocalized states whereupon they travel large distances before relaxing back down to lower states. Adapted from ref (34). Copyright 2021 The Authors.

We note that this behavior, where the exciton wave function’s spatial extent and energy fluctuate in time, is similar to the coherent *intramolecular* exciton-vibrational dynamics shown previously for excitons residing on a single molecule. Key differences, however, are the randomness with which excitons access more delocalized states and the fact that this can occur at all points in the exciton’s lifetime.^38,39^ These dynamics also interestingly bare many similarities to the dynamics demonstrated for *charges*, and a similar (and successful) transient charge (de)localization scenario has been well-established, as will be discussed later.^28,31,33,40,41^

It is instructive to consider more closely the details of the simulations. In them, the nanofibers were coarse-grained as a one-dimensional series of sites, with the excitons modeled by solving the time-dependent Schrödinger equation and the vibrational modes treated classically, while accounting explicitly for the exciton–phonon interactions (namely, excitonic back forces on the nuclei that drive the formation of exciton-polarons were included). These lattice fluctuations drive the system close to the crossing seam between different adiabatic potential energy surfaces, which allows for the stochastic nonadiabatic hopping of excitons between these surfaces (for full details, see refs (34), (42), and (43)). The site-to-site couplings that facilitate transport are the Coulombic interactions (computed using a multicentric expansion of the transition dipoles, i.e., using interacting transition charges). These couplings decay slowly with interchromophore distance, *d*, reaching the Förster *d*^–6^ dependence at large . By combining the results from multiple individual exciton trajectories (see Figure 2d,e), the simulations resulted in *D* values of 0.2 and 6 × 10^–4^ cm^2^/s, when accounting for and ignoring long-range couplings, respectively. This first value compares well with the experimental value of 1.1 cm^2^/s (note that a purely FRET-based model gives a value of 2 × 10^–3^ cm^2^/s), and so this was considered evidence that the simulations provided a reasonably accurate physical picture of the underlying physics at play. Further investigation of single-exciton trajectories revealed that the excitons periodically exchanged energy with vibrational modes to access higher-energy states with higher inverse-participation ratio (IPR) values; IPR is a measure of how many molecules/sites an exciton is delocalized over. Crucially, when the excitons reached these delocalized states, they tended to move large distances as shown in Figure 2f,g. This gives rise to the schematic shown in Figure 2h where the exciton sporadically gets kicked up to more delocalized/spatially extended states whereupon it “surfs” along the excitonic density of states (EDOS) before falling back down to a more localized state again. This motivates the term “transient delocalization”, as while the exciton is mostly localized on a single chromophore, it occasionally reaches more delocalized states. In a sense, this behavior combines qualitative elements of both FRET and coherent transport; each jump could be considered simplistically on the broader scale as a “hop”, with this process mediated by the evolution of the excitons when they reach delocalized states.

This emergent transient delocalization behavior is surprising, particularly given the large body of past work studying exciton transport in P3HT-based systems, and we must ask ourselves what is special about the living CSDA-derived P3HT nanofiber films as opposed to the traditional spin-coated P3HT thin films? At one level, the nanofibers are highly pure; living CDSA naturally lends itself to excluding imperfect polymer chains with bends, kinks, or twists, and evidence shows that the nanofibers exhibit very low energetic order near the band edge with an Urbach energy of *E*_u_ = 29 meV, together with an absence of deeper lying traps. This will prevent excitons from being either weakly or strongly trapped at shallow or deep traps. In the simulations, *D* is moderately dependent on energetic order, with *D* increasing or decreasing by ∼40% when the inhomogeneous broadening was halved or doubled, respectively. But given that spin-coated P3HT had an Urbach energy of 53 meV, this means the enhancement in *D* is not well-explained by energetic order alone. In fact, the nanofibers possess another property which appears to be vital for transient delocalization: their structure promotes long-range dipole–dipole couplings.

The role of long-range couplings was studied in more detail in a follow-up study by Prodhan et al., who repeated the simulations on the same nanofiber system under a broader variety of model conditions (see Figure 3a).^42^ The most dramatic effects were seen when varying the long-range couplings either by artificially controlling the allowed interaction range between chains or by considering much shorter “6-mer” chains which contain 6 polythiophene units. These shorter chains have stronger couplings at close range but much weaker couplings at long-range (see Figure 2c). An intuitive explanation of this can be found by approximating excitons on the chains as simple electric dipoles the length of the chain; in this case, the electric field lines and hence Coulombic couplings for shorter chains will be reduced (increased) at long (short) range perpendicular to the chain/dipole. This end result is that as shown in Figure 3c: the 6-mer has a  that is an order of magnitude lower than that of the 30-mer. We also see that when just considering the 30-mer, the  plummets to a very low value of 0.0006 cm^2^/s when the long-range couplings are not included in the simulations. These results demonstrate that transient delocalization can be critically dependent on the presence of long-range couplings, which itself is greatly assisted by long-range structural order (which enables long interaction ranges) and long intramolecular coherence lengths. This helps to reconcile the lack of observations of this type of behavior in previous (semicrystalline) solution-processed polymer thin films, P3HT or otherwise, as such films have pervasive structural disorder which retards the interaction range, and kinks or twists in the polymer disrupt the exciton’s intramolecular coherence length, lowering long-range couplings.

**Figure 3:**
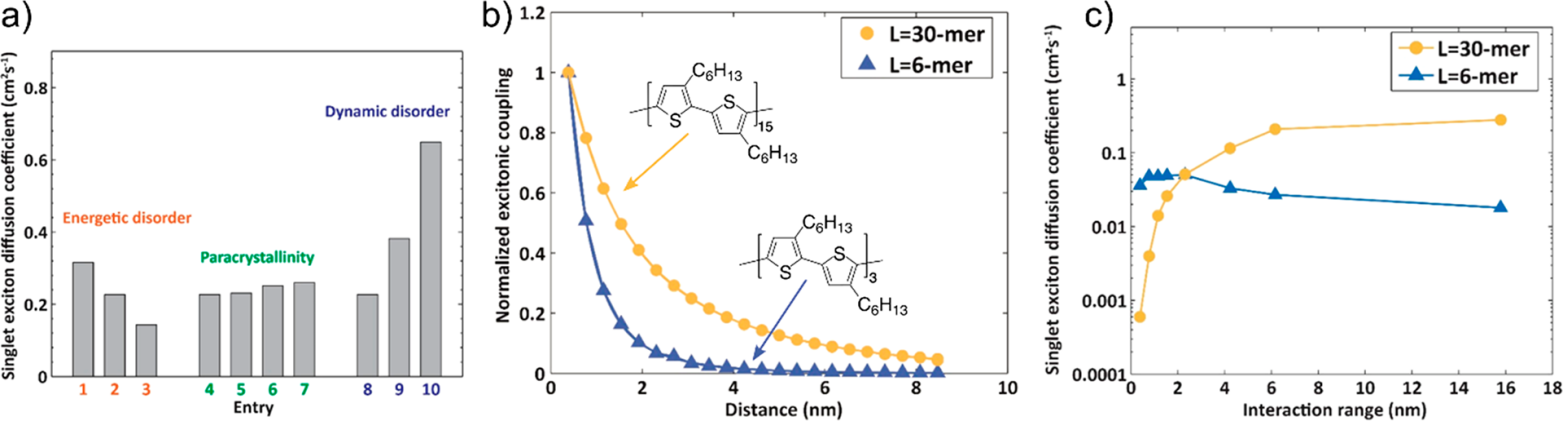
(a) Variation of *D* in P3HT nanofibers due to various disorders: entries 1–3 are for energetic disorders of 18, 36, and 72 meV; 4–7 are for paracrystalline disorders of  = 0%, 1%, 10%, and 20%; and 8–10 for dynamic disorders of  = 0, 0.5, and 1, respectively, where  is the variance of the thermal distribution and  is the thermal average of the squared couplings.  is relatively insensitive to static and positional disorders and increases only modestly when including dynamic nonlocal exciton–phonon interactions. This increase in  with off-diagonal disorder likely comes about because it gives more fluctuations in the exciton’s energy and more “chances” to reach the higher-lying mobile states. (b) Distance-dependent excitonic couplings in P3HT nanofiber for 6-mer versus 30-mer, with biexponential fits. The 30-mer has weaker nearest-neighbor couplings (9 meV) than the 6-mer (65 meV), but it has much stronger couplings at long-range. (c) Dependence of *D* on range of excitonic couplings included in simulations for 6-mer and 30-mer, where *D* is critically dependent on both the interaction range of the couplings and the length of the unimer. Adapted with from ref (42). Copyright 2021 American Chemical Society.

A further development in the application of nonadiabatic molecular dynamics methods to molecular semiconductor systems is given by the work of Giannini et al.^43^ Here, the authors study the evolution of excitons in several OSCs ranging from anthracene to the nonfullerene acceptor, Y6. These molecular systems emphasize short-range interactions in the same way the 6-mer does. Importantly, the simulations predict that transient delocalization is indeed present to varying degrees in each OSC, as analysis of individual trajectories reveals the excitons occasionally reach higher-energy delocalized states whereupon they move large distances. In one OSC, dicyanovinyl-capped S,N-heteropentacene (DCVSN5), the exciton can occasionally reach large IPR values of 5–12, and access to such states is associated with large movements in space. This results in a relatively large  of 0.06 cm^2^/s for DCVSN5. For Y6, a material used in many high-performing photovoltaics, an even larger  of 0.15 cm^2^/s was found, a value comparable to that of the P3HT nanofibers. For anthracene, while transient delocalization was indeed present, the maximum IPR in single trajectories stayed below ∼2, and accordingly this was associated with small movements of the exciton and eventual *D* values of 3.3 × 10^–3^ and 0.77 × 10^–3^ cm^2^/s depending on the crystal axis. Overall, the calculated *D* values compared favorably to available experimental values.

These works suggest that transient delocalization is a general phenomenon that occurs in ordered OSCs. The key question then appears to be to *what degree* is transient delocalization present. Clearly, for anthracene its effect is rather minimal; that is, the exciton does not exhibit very large fluctuations in delocalization, and so the behavior could be approximated by modeling the exciton as localized on a single chromophore with its movement incoherent. In such a case, FRET will be an appropriate approximation. However, for materials such as Y6 and the P3HT nanofibers, transient delocalization plays a much larger role with frequent and large fluctuations in the excitons’ IPR, and it is essential for describing the transport.

*Emerging Design Rules and Open Questions*. With the evidence now that transient delocalization can exist in ordered OSCs, and that it can lead to long-range exciton transport, it is worth hypothesizing what potential material design rules exist for effective transient delocalization so that we may develop it further. Note that the following suggested rules are somewhat speculative as the investigation of transient delocalization is still in its infancy, and much more research is called for in this area.

To begin, we must first remind ourselves of the key player in the transient delocalization scenario: the higher-lying delocalized, or “*mobile*”, exciton states, which play an *outsized* role in transport in comparison to the lower-lying states. It follows that engineering effective transport should be contingent on increasing frequency of access to these mobile states, the duration of their occupancy, and their spatial extent/ability to transfer excitons over large distances. One way of achieving this is by enhancing the long-range electronic couplings within the system. As was seen in the P3HT nanofibers, increased long-range couplings in the 30-mer lead to an order-of-magnitude enhancement in  over the 6-mer. This was explained by the long-range couplings increasing the spatial extent of the higher-lying states and through the rationale that they facilitated access to these states.^42^ A design rule may therefore be to optimize long-range couplings in OSCs via strategies such as increasing structural order or increasing the “size” of the exciton and its dipole to distribute exciton couplings to molecules further away. This latter factor is particularly interesting as it motivates a new synthetic space where the design and arrangement of chromophores can be tuned to optimize for long-range interactions, or more broadly, unconventional patterns of exciton couplings within OSCs that may potentially promote transport.

Another possible design rule is given by the correlation observed by Giannini et al. between the average IPR and  over a range of molecular OSCs.^43^ In other words, the more delocalized an exciton is on average, the faster it will diffuse. This trend, however, was not strictly observed for all the OSCs investigated, nor was it observed for the P3HT nanofibers, with the 30-mer having a slightly lower average IPR than the 6-mer despite a higher . These inconsistencies can be rationalized by the fact that the average IPR will mainly be predicated by the lower-lying states because they will be the ones most often occupied, and hence, it is not a direct indicator of the nature of the all-important higher-lying mobile states. Hence, average IPR can be seen only as a partial or weak predictor of , or by extension, transient delocalization.

Energetic disorder also plays a significant role, and candidate OSC materials for transient delocalization should be engineered to minimize energetic disorder and deep sub-bandgap states which deeply trap excitons, preventing them from reaching the mobile states. The reorganization energy also appears to play an important role and was also observed to be correlated with .^43^ This energy can be thought of as the activation energy barrier for exciton transfer from one chromophore to another—a result of the exciton distorting a chromophore’s bond lengths and geometry. Clearly, lower reorganization energies are greatly preferable, and a suggested target is to have the reorganization energy smaller than two times the excitonic couplings, at which point excitons become considerably more delocalized and free to diffuse.^43^

Another crucial feature of OSCs that enables transient delocalization is the exciton–phonon couplings, as they facilitate access to the mobile states. This is particularly interesting as dynamic disorder is normally considered destructive for the formation and hence utilization of delocalized states. Instead, as seen in the case of the P3HT nanofibers, these delocalized states still exist at higher energies, and dynamic disorder is what allows excitons to reach those states. This may be initially surprising because the main vibrational mode of P3HT, the C=C stretch at 1450 cm^–1^, is not thermally occupied at room temperature because of Boltzmann statistics. Yet, this mode still exhibits zero-point motion which introduces periodic fluctuations in the exciton’s energy, which, along with the thermally occupied modes, is sufficient to allow the exciton to cross over to temporarily degenerate delocalized states. Overall, this is suggestive of a paradigm shift in the way excitons are considered in bulk OSCs, especially in the context of exciton transport, with excitons usually pictured as static entities that do not fluctuate in energy. The reality is that dynamic disorder results in constant fluctuations in the exciton’s energy, allowing the excitons to cross over to different adiabatic surfaces with different spatial extents, which can allow for highly efficient transport under the right conditions. This notion of exciton transport in bulk OSCs is more in line with the literature on natural LHCs, where it has long been recognized that energy fluctuations from vibrations play a key role in mediating the overall evolution of the system.^9,19,44^

The exact role of exciton–phonon couplings still requires much more investigation, however, and their overall importance may depend on the interaction of several factors, including energetic disorder, electronic couplings, and reorganization energies. For example, when sources of disorder are high, phonon couplings may be desirable to overcome energetic barriers, but when these barriers are vanishing, the couplings may play a more harmful role as they introduce dynamic disorder which now becomes the dominant form of disorder.^43^ The phonons could also have more inherently “quantum” effects; for example, in some cases they have been shown to promote electronic coherences, which could be spontaneously created for short periods of time when the exciton sporadically reaches up into the higher-lying states.^45^ More research is called for into this topic, particularly into the often-overlooked role of zero-point motion, as well as factors such as the anharmonicity of vibrations which becomes important for low-frequency modes.^46,47^ We note that a highly interesting subject will be the experimental measure of the temperature dependence of transient delocalization in comparison to theory across a range of systems.

Other important topics include more experimental validations of transient delocalization, particularly at lower excitation densities to fully avoid annihilation-based effects, and further refinement of the simulations, e.g., by inclusion of more vibrational modes.^48^ An exciting direction is to test in what other material systems transient delocalization may occur, and especially good candidates for this are other aligned supramolecular systems like the P3HT nanofibers which favor long-range interactions,^6^ as well as materials which promote delocalization such as nonfullerene acceptors. A comparison of H- vs J-aggregates would also be of great interest. In H- (J-) aggregates, the dipoles in the solid are aligned cofacially (end-to-end), leading to a reduction (increase) in the oscillator strength near the band edge in comparison to states higher in energy in the band.^49,50^ Because higher oscillator strength is correlated with stronger electronic couplings between chromophores, we may expect more delocalized states *closer* to the band edge as was seen in J-aggregate porphyrin nanotubes versus the case of the mixed H/J-type P3HT nanofibers where those delocalized states are higher in energy.^7^ Further investigation of the impact of these differences on transient delocalization is certainly warranted. Finally, we remark that from a fundamental point of view, it would be interesting to compare transient delocalization to other phenomena related to disorder.

*Comparison between the Transient Delocalization of Excitons versus Charges*. We have seen that in transient delocalization, higher-lying delocalized states can play a key role in mediating the transport of excitons. However, it is interesting and instructive to note that these states have been shown to play a key role in other contexts too. For example, they have been shown to facilitate ultrafast charge separation at heterojunctions, where delocalized π-electron states in the acceptor allow electrons to rapidly propagate away from the heterojunction.^51,52^ Much work has also detailed the role of higher-lying states in charge transport within a single OSC material. In fact, it is now well-established that this leads to the equivalent transport mechanism of transient *charge* (de)localization, a cousin of transient (exciton) delocalization.^28,31,33,40,41^

Transient charge and exciton delocalizations are, unsurprisingly, quite similar; in the case of charges, the carriers constantly undergo large fluctuations in their delocalization extent due to phonons, with access to delocalized states associated with large movements of the carrier. For highly conductive crystals such as pentacene, the carrier can become transiently delocalized over tens of molecules, as was nicely visualized by Giannini et al. (see Figure 4c–f).^28^ However, a key difference between the two comes from the fact that while excitons can have long-range couplings between molecules due to dipole–dipole interactions, charges are limited to nearest-neighbor interactions because they rely on direct wavefunction overlap. These nearest-neighbor interactions are much stronger in the case of charges and can be >100 meV in comparison to ∼10 meV for the P3HT nanofibers. In this sense, the “structure” of the Hamiltonian is more akin to that of the hypothetical 6-mer and molecular OSCs. Interestingly, the 6-mer and molecular OSCs exhibit a “smoother” IPR evolution than the 30-mer (Figures 4a,b), with the 6-mer’s evolution more closely resembling that of the charge carriers for p-MSB and pentacene (Figures 4c,d).

**Figure 4:**
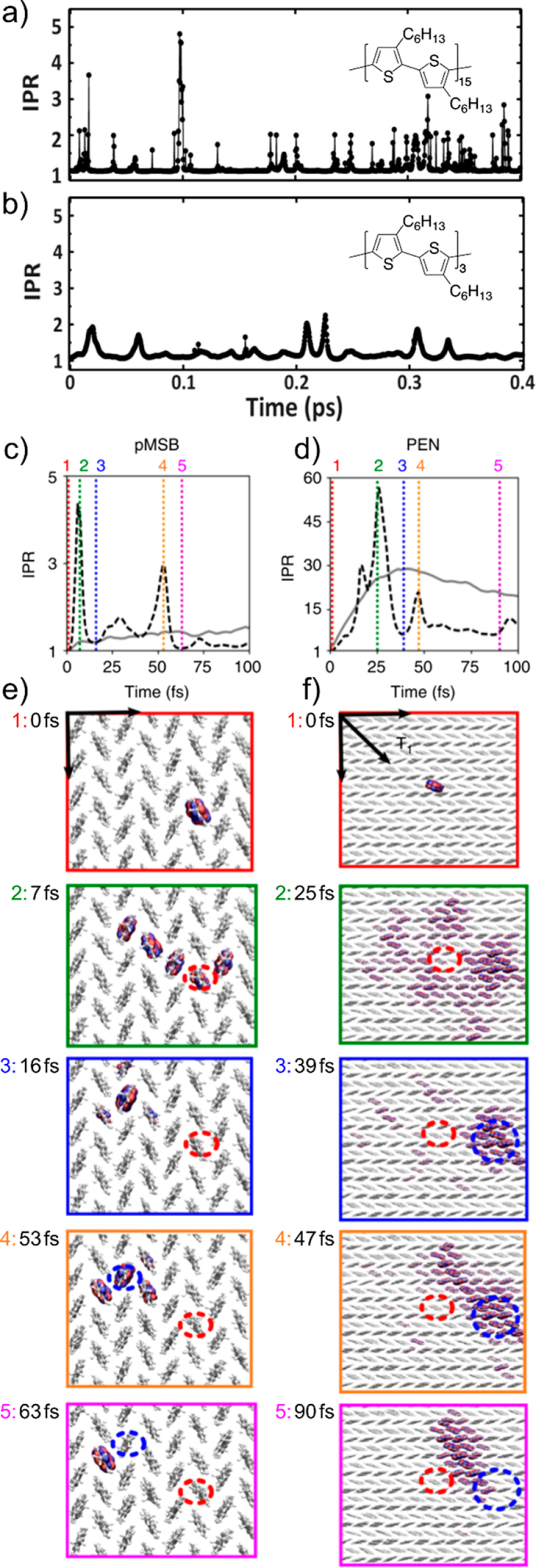
Transient exciton delocalization and transient charge (de)localization. (a and b) Representative IPR traces of 30-mer and 6-mer, respectively, showing the smoother evolution of the IPR in the case of the 6-mer where short-range dipolar interactions dominate. Adapted from ref (42). Copyright 2021 American Chemical Society. (c and d) IPR traces of charge carrier in pMSB and pentacene in representative FOB-SH trajectories, respectively (black dashed lines). Gray solid lines are average of 300 trajectories. The charge IPR traces are broadly similar to those of the excitons’, particularly when compared to the 6-mer. (e and f) Several associated snapshots of hole carrier wave function in the pMSB and pentacene crystals, respectively. The charge carrier occasionally experiences large fluctuations in its spatial extent, with these fluctuations visualized dramatically in the case of pentacene. Adapted with permission from ref (28). Copyright 2019 The Authors.

Given that charges rely on short-range interactions, it is interesting to compare the role of off-diagonal energetic disorder. In particular, it is known that this disorder, which originates from thermal fluctuation of the site-to-site electronic couplings separately, has a large impact on charge mobility^28,31,53^ but appears to be less crucial for excitons. This is understandable given that vibrations will not make much difference to the dipole–dipole couplings between molecules far from one another, but at short-range, they can play a critical role in potentially misaligning orbitals and strongly affecting coupling.^53^ Diagonal energetic order—the static or dynamic fluctuation of the site energies themselves—also may be less critical for excitons, as these fluctuations may be “averaged” out by the fact that the excitons are coupled to many different sites. We also note the differences in reorganization energies for exciton and charge transfer. Because exciton transfer involves *both* the depopulation of the HOMO and population of the LUMO, it results in larger changes to the overall bonding character than charge transfer where just one of the changes occurs. This gives larger reorganization energies for exciton transfer, resulting in overall lower average IPR values and levels of delocalization.^43^ Finally, structural order and long-range interactions appear to be crucial to excitons but not for charges. In fact, many very high-mobility materials exhibit a near-amorphous microstructure (note that they do have very low energetic disorder).^54^ Such materials do not perform well for exciton transport. Thus, while transient delocalizations in the cases of excitons and charges bear similarities, it appears that the material design rules are different in each case, and any given material may not necessarily exhibit *appreciable* amounts of both phenomena. It should be noted, however, that this subject needs more investigation and that many of the comparative assertions are somewhat speculative.

Finally, we note that in some materials there may not be as clear of a distinction between traditional Frenkel-type excitons and free charges, with significant charge-transfer (CT) character contributing to the exciton. This could have important consequences. For example, coupling to CT excitons in oligoacene crystals was shown to be key for reproducing experimental absorption values, and it greatly enhanced the curvature near the lower band minimum, resulting in ∼6× higher calculated diffusion coefficients in tetracene when CT states were included, with similar effects in anthracene and pentacene.^55^ We also note that strictly CT states themselves have been shown to be relatively mobile (5–10 nm diffusion lengths), with the transport mechanism suggested to be unique in that the electron–hole spacing may vary over time, which could have significant implications for the transport.^56^ For instance we may speculate that if the hole and electron can move independently to some degree, and if their separation has a significant effect on site-to-site electronic couplings, then factors critical to charge transport, e.g., off-diagonal energetic order, may now become important for exciton transport. Again, investigation of the influence of CT character on exciton transport and transient delocalization is expected to be an interesting direction of study.

In summary, transient delocalization presents itself as a new mechanism of transport for moderately delocalized excitons in ordered OSCs. Importantly, it allows for efficient long-range transport and can occur in device-relevant materials, and so engineering transient delocalization may provide a path to improve device efficiencies and may ultimately allow for new design architectures. At the fundamental level, transient delocalization also forces us to revisit the prevailing physical picture of excitons, particularly in device-relevant bulk OSC materials, where excitons are often pictured as static entities. Instead, strong exciton–phonon couplings in organic materials—even from non-thermally occupied modes—introduces large constant fluctuations in the exciton’s energy and spatial extent that can result in unexpected phenomena, such as efficient exciton transport.

There are still many open questions, however, and exploration of transient delocalization’s interplay with factors such as dynamic and static disorder, reorganization energies, and electronic couplings in different material systems is expected to be highly fruitful. On the broader scale, it will also be fascinating to see whether transient delocalization is limited to just organic materials; the idea of transient delocalization is in fact very general: exciton–phonon couplings allow excitons to temporarily access higher-lying delocalized states which can have an outsized effect on transport, and so further work could explore this possibility in other excitonic systems, such as two-dimensional perovskites and transition-metal dichalcogenides.
